# Triple Combination of Amantadine, Ribavirin, and Oseltamivir Is Highly Active and Synergistic against Drug Resistant Influenza Virus Strains *In Vitro*

**DOI:** 10.1371/journal.pone.0009332

**Published:** 2010-02-22

**Authors:** Jack T. Nguyen, Justin D. Hoopes, Minh H. Le, Donald F. Smee, Amy K. Patick, Dennis J. Faix, Patrick J. Blair, Menno D. de Jong, Mark N. Prichard, Gregory T. Went

**Affiliations:** 1 Adamas Pharmaceuticals, Inc., Emeryville, California, United States of America; 2 Utah State University, Logan, Utah, United States of America; 3 Naval Health Research Center, San Diego, California, United States of America; 4 Academic Medical Center, University of Amsterdam, Amsterdam, The Netherlands; 5 University of Alabama, Birmingham, Alabama, United States of America; Washington University, United States of America

## Abstract

The rapid emergence and subsequent spread of the novel 2009 Influenza A/H1N1 virus (2009 H1N1) has prompted the World Health Organization to declare the first pandemic of the 21^st^ century, highlighting the threat of influenza to public health and healthcare systems. Widespread resistance to both classes of influenza antivirals (adamantanes and neuraminidase inhibitors) occurs in both pandemic and seasonal viruses, rendering these drugs to be of marginal utility in the treatment modality. Worldwide, virtually all 2009 H1N1 and seasonal H3N2 strains are resistant to the adamantanes (rimantadine and amantadine), and the majority of seasonal H1N1 strains are resistant to oseltamivir, the most widely prescribed neuraminidase inhibitor (NAI). To address the need for more effective therapy, we evaluated the *in vitro* activity of a triple combination antiviral drug (TCAD) regimen composed of drugs with different mechanisms of action against drug-resistant seasonal and 2009 H1N1 influenza viruses. Amantadine, ribavirin, and oseltamivir, alone and in combination, were tested against amantadine- and oseltamivir-resistant influenza A viruses using an *in vitro* infection model in MDCK cells. Our data show that the triple combination was highly synergistic against drug-resistant viruses, and the synergy of the triple combination was significantly greater than the synergy of any double combination tested (*P*<0.05), including the combination of two NAIs. Surprisingly, amantadine and oseltamivir contributed to the antiviral activity of the TCAD regimen against amantadine- and oseltamivir-resistant viruses, respectively, at concentrations where they had no activity as single agents, and at concentrations that were clinically achievable. Our data demonstrate that the TCAD regimen composed of amantadine, ribavirin, and oseltamivir is highly synergistic against resistant viruses, including 2009 H1N1. The TCAD regimen overcomes baseline drug resistance to both classes of approved influenza antivirals, and thus may represent a highly active antiviral therapy for seasonal and pandemic influenza.

## Introduction

Globally, influenza viruses cause substantial morbidity and mortality in humans and economic dislocation in afflicted nations. Each year in the United States, seasonal influenza virus infection result in an estimated 36,000 deaths and 200,000 hospitalizations [1]. Antiviral drugs are an important means to mitigate the impact of the yearly influenza epidemics and potential pandemics. Currently, two classes of antiviral drugs have been approved for the prevention and treatment of influenza infection – the M2 channel inhibitors (aminoadamantanes; amantadine and rimantadine) and the neuraminidase inhibitors (NAIs; oseltamivir and zanamivir). However, the prevalence of drug-resistant strains, which has been reported for both classes of antiviral drugs for seasonal influenza [2], [3], could undermine their clinical benefit when utilized as monotherapy. Indeed, in 2009, the Centers for Disease Control and Prevention (CDC) reported that 100% of the seasonal H3N2 virus isolate tested were resistant to the adamantanes, and 99.6% of the seasonal H1N1 viruses tested were resistant to oseltamivir [4].

In April, 2009, a novel H1N1 virus of swine-origin capable of human-to-human transmission likely emerged in Mexico and was first isolated from patients enrolled in two separate surveillance activities in Southern California [5]. The emergence and spread of 2009 H1N1 to over 168 countries has led U.S. officials [6] and the World Health Organization (WHO) [7] to declare a public health emergency; on June 11, 2009 the WHO raised the influenza pandemic alert from phase 5 to phase 6, the official declaration of a pandemic [8]. Early published results from the CDC showed that 2009 H1N1 bears the amantadine-resistance associated S31N mutation in the M2 ion channel, but remains susceptible to oseltamivir and zanamivir [9]. More recently, however, the CDC has reported that ten 2009 H1N1 isolates tested in the United States have been found to be resistant to oseltamivir, raising the concern that dually resistant viruses may become prevalent [4].

In an earlier study, we explored the *in vitro* antiviral activity and synergy of single, double, and triple combinations of amantadine, ribavirin and oseltamivir against a panel of influenza A viruses that were susceptible to these drugs [10]. Our hypothesis was that a triple combination antiviral drug (TCAD) regimen composed of drugs with different mechanisms of action, and which act at different stages in the viral life cycle, could result in synergistic antiviral activity. Our results showed that these drugs did indeed act synergistically, with the triple combination showing significantly greater synergy than any of the double combinations evaluated. Furthermore, we found that the synergy of the TCAD regimen was maintained across multiple seasonal and avian influenza A strains, including the three major subtypes – A/H1N1, A/H3N2, and the avian A/H5N1 – that currently cause significant morbidity and mortality in humans.

In this study, we sought to evaluate the activity and synergy of the TCAD regimen against influenza viruses which were resistant to amantadine or oseltamivir. Our data showed that against amantadine-resistant viruses – both seasonal and 2009 H1N1 – and oseltamivir-resistant seasonal viruses, the TCAD regimen was strongly synergistic, and the synergy of the TCAD regimen was greater than the synergy of any double combination. Surprisingly, we found that amantadine and oseltamivir contributed to the synergy of the TCAD regimen at concentrations where they had no activity as single agents, and at concentrations that were clinically achievable. Our findings highlight the utility of the TCAD regimen as a potential approach to address the current limitations of antiviral potency and drug resistance, and as a viable broad-spectrum therapeutic option for serious influenza virus infection.

## Materials and Methods

### Antiviral Compounds

Oseltamivir carboxylate, the active metabolite of oseltamivir, was obtained from Charnwood Molecular (Loughborough, U.K.) through synthesis via the NBoc-protected acid from oseltamivir phosphate. Amantadine was obtained from Moehs Catalana (Barcelona, Spain). Ribavirin and rimantadine were purchased from Sigma-Aldrich (St. Louis, MO). Peramivir was obtained from Jubilant Chemsys LTD (Uttar Pradesh, India) as free base through NBoc synthesis, and purity was confirmed using NMR and chiral HPLC. Zanamivir was obtained from Haorui Pharma-Chem, Inc. (Edison, NJ).

### Influenza Viruses

Two 2009 H1N1 strains – influenza A/California/05/09 (H1N1) and influenza A/California/10/09 (H1N1) – were obtained from the Naval Health Research Center as the result of ongoing influenza surveillance studies. A third 2009 H1N1 strain, A/California/04/10 (H1N1) was received from the Centers for Disease Control and Prevention (CDC, Atlanta, GA). Influenza A/New Caledonia/20/99 (H1N1) was provided by the CDC, and the amantadine-resistant V27A mutant was generated by passaging in Madin-Darby canine kidney (MDCK) cells in the presence of amantadine. Influenza A/Duck/1525/81 (H5N1) was provided by Dr. Robert Webster (St. Jude Children Research Hospital, Memphis, TN), and the A30T amantadine-resistant mutant was generated by passaging in MDCK cells in the presence of amantadine. The oseltamivir-resistant influenza A/Mississippi/3/01 (H1N1) [H274Y] was provided by the Neuraminidase Inhibitor Surveillance Network (Melbourne, Australia), and the oseltamivir-resistant A/Hawaii/21/07 (H1N1) was kindly provided by Dr. Larisa Gubareva (CDC, Atlanta, GA). The viruses were passaged in MDCK cells (American Type Culture Collection, Manassas, VA) to create working stocks, which were used for the antiviral assays. Additionally, the genotype of the matrix protein 2 (M2), hemagglutinin (HA), and neuraminidase (NA) for each virus were confirmed by Sanger sequencing.

### Cells and Growth Medium

Cells were passaged in minimal essential medium containing 5% fetal bovine serum (Hyclone Laboratories, Logan, UT). During antiviral evaluations, the serum was removed and the medium was supplemented with gentamicin (50 µg/mL), porcine trypsin (10 units/mL) and EDTA (1 µg/mL).

### Cell-Based Assays

To obtain single agent concentration-response curves, individual drugs were added to MDCK cells in 96-well microplates (8×10^4^ cells/well) using three wells for each concentration used. The compounds were added at the following concentrations: oseltamivir carboxylate, zanamivir, and peramivir at 0, 0.000032, 0.0001, 0.00032, 0.001, 0.0032, 0.01, 0.032, 0.1, 1.0, 10.0 and 100 µg/mL; amantadine, rimantadine, and ribavirin at 0, 0.001, 0.0032, 0.01, 0.032, 0.1, 0.32, 1, 3.2, 10, 32 and 100 µg/mL. Untreated wells of infected cells (virus controls), uninfected cells (cell controls), and drug cytotoxicity controls (cells and drugs only, using the same dilution range for each drug as the test wells) were included on each test plate. At three days after infection, the virus control wells exhibited 100% cytopathology. The 50% effective concentration (EC_50_) and 50% cytotoxic concentration (TC_50_) was determined for each drug as outlined below.

For double and triple combination studies, each drug was tested in triplicate at five or six concentrations (including no drug), in which the high concentration for each drug was set to approximate the EC_50_ of the drug as a single agent. The concentrations of each drug used in double and triple combinations against each virus are provided in Table S1. The cytotoxicity of the double and triple drug combinations were determined using the same experimental format in three separate experiments, and using the same concentration ranges as outlined in Table S1.

The extent of viral cytopathology in each well was determined by staining with Neutral Red (NR) as detailed elsewhere [11]. Briefly, the cells were stained with 0.011% NR diluted in MEM to determine cell viability. Two hours later the plates were processed for quantification of NR uptake into viable cells. The amount of NR taken up by cells was determined spectrophotometrically.

### EC_50_, TC_50_, and Synergy Calculations

EC_50_, TC_50_, and synergy calculations were done as described previously [10]. Briefly, EC_50_ and TC_50_ calculations for single agents were made by normalizing the NR data for each well against the cell and virus controls, which was assumed to represent 100% cell viability and 0% cell viability, respectively. Normalized data were plotted as percent cell viability versus compound concentration. The data points were then fitted using four-parameter curve fitting in Graphpad Prism (Graphpad Software, La Jolla, CA) to derive the EC_50_ and TC_50_. Statistical comparisons between best-fit EC_50_ values for any two curves were performed in Prism using the extra sum-of-squares F test; differences in EC_50_ values between two curves with a *P*-value of <0.05 were considered significant.

Synergy was calculated using the MacSynergy II software developed by Prichard and Shipman, which was modified to accommodate a three-drug combination [12] and is similar to that reported previously describing this approach [13]. The theoretical additive interactions were calculated from the concentration-response curves of each drug as a single agent. This calculated additive surface was then subtracted from the observed, experimental surface to reveal regions that deviate from the calculated additive effects. Purely additive interactions are represented graphically as areas in grey, indicating that they do not differ from the calculated additive effects. Synergistic interactions result in greater inhibition than the calculated inhibition, and are represented as areas in blue. Conversely, antagonism is represented as areas in red. Synergy plots are shown as the percent inhibition above or below expected (calculated additive inhibition), and are presented as the mean of three replicates at a level of 95% confidence, which eliminates insignificant deviations from the additive surface.

Synergy volume for each double and triple combination was also calculated, which represents the sum of the synergy or antagonism across all concentrations of a combination. Synergy volumes are presented as a quantitative measure of the overall interaction of the drugs within a combination. As determined by cytopathic effect (Neutral Red assay), synergy volumes >100 µg/mL^2^% for double combinations or >100 µg/mL^3^% for triple combinations are considered to be strongly synergistic. Conversely, combinations with synergy volumes <−100 µg/mL^2^% or µg/mL^3^% are considered to be strongly antagonistic.

The synergy of the cytotoxic effects of double and triple drug combinations were calculated in a similar manner.

### Statistical Analysis of Synergy Volumes

Between three and nine independent experiments were conducted using identical dosing ranges for amantadine, ribavirin, and oseltamivir carboxylate against each virus. The experiments used a 3-replicate plate format, for a total of 9 to 27 observations for each condition. The data from the independent experiments were merged and subjected to statistical analysis using the random effects model. The raw data were imported into the program R and were normalized to the virus and cell controls as described above, and synergy (percent inhibition above calculated) is calculated as above. For synergy volume estimates, random effects models were applied to the data to account for variation between replicate measures within experiments. Therefore, the models took the form as given by:
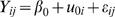
where Y_ij_ is the measure of the j^th^ replicate in experiment i; β_0_ is the grand mean; u_0i_∼N(0,τ^2^); and ε_ij_∼N(0,σ^2^). As the observations in the data are not independent, this model allowed for proper estimation of standard errors. The standard errors were then used to determine the statistical significance of synergy volume.

### Determination of Inhibitory Quotient

The inhibitory quotient (IQ) is defined herein as the ratio of the expected average total (free and protein-bound) plasma concentration (C_ave_) of each drug at recommended dosage to the EC_50_ (C_ave_/EC_50_). Thus, an IQ of 1 or greater means that the achievable total plasma concentration of the drug is equal to or greater than the *in vitro* 50% effective concentration, and the higher the IQ translates to a greater predicted efficacy. The C_ave_ of each drug was determined by pharmacokinetic modeling using a non-compartmental model in the progam WinNonLin. The recommended doses, along with the pharmacokinetic parameters, used for modeling were obtained from the package inserts for amantadine [14], oseltamivir [15], and ribavirin [16], and the references therein. For ribavirin, since the plasma concentration does not achieve steady-state until ∼4 weeks after the start of dosing, the C_ave_ was determined for the first 10-day window. Based on these parameters, the expected C_ave_ for amantadine was determined to be 0.43 µg/mL based on the recommended dosage of 100 mg twice daily for the treatment of influenza infection; the expected C_ave_ for oseltamivir was determined to be 0.3 µg/mL based on the recommended dosage of 75 mg twice daily for the treatment of influenza infection; and the expected C_ave_ of for ribavirin was determined to be 1.3 µg/mL after 10 days of treatment based on the recommended dosage of 600 mg twice daily for the treatment of hepatitis C infection. To determine the IQ of the triple combination, amantadine, ribavirin, and oseltamivir carboxylate were tested as a fixed ratio combination, wherein the ratio of the three drugs was kept constant even as the total concentration of drugs varied. The ratio of drugs in the TCAD regimen was based on the expected C_ave_ of each drug. A dilution curve of TCAD regimen was created by first preparing a solution of all three drugs at 100-fold the C_ave_ of each drug (43 µg/mL amantadine, 30 µg/mL oseltamivir carboxylate, and 130 µg/mL ribavirin), and then serially diluting this solution in 0.5-log_10_ increments. In this manner, the EC_50_ of TCAD regimen as a fixed dose combination was determined as ratio of the C_ave_ and expressed in units of fold-C_ave_.

## Results

### Activity of Antiviral Drugs as Single Agents against 2009 H1N1

The susceptibility of three 2009 H1N1 influenza strains – A/California/04/09 (CA04), A/California/05/09 (CA05) and A/California/10/09 (CA10) – to each of six antiviral drugs (amantadine, rimantadine, oseltamivir carboxylate, zanamivir, peramivir, and ribavirin) as single agents was determined by measuring the inhibition of virus-induced CPE in MDCK cells. Against the three strains, oseltamivir carboxylate, zanamivir, peramivir, and ribavirin produced concentration-dependent inhibition of cytopathic effect (data not shown). Amantadine was active only at higher concentrations (EC_50_ of 16–20 µg/mL; 85–106 µM) (Table S2), which represents a >250-fold reduction in susceptibility compared to the published values for a wild-type virus[17]. Rimantadine did not produce inhibition up to the 50% cytotoxic concentration (EC_50_>12 µg/mL; >55 µM). The EC_50_ values for the six drugs as single agents against all three strains are summarized in Table S2, and confirm that the three virus strains remain susceptible to oseltamivir carboxylate, zanamivir, peramivir, and ribavirin. These results are consistent with the results previously published that demonstrated that 2009 H1N1 contained a mutation (S31N) in the M2 channel that has been associated with resistance to adamantanes, but remained susceptible to NAIs [18].

### Synergy of Double and Triple Combinations of Amantadine, Ribavirin, and Oseltamivir Carboxylate against 2009 H1N1

**Figure 1:**
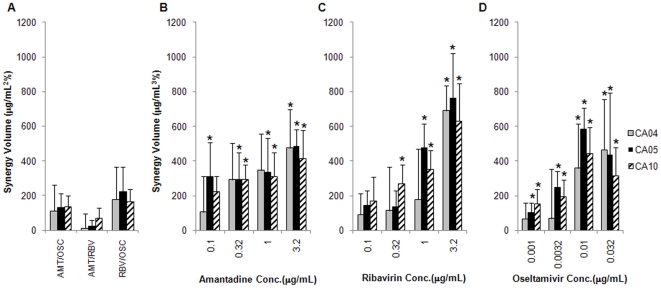
Synergy of double and triple combinations of amantadine, ribavirin, and oseltamivir carboxylate against 2009 H1N1. Amantadine-resistant 2009 H1N1 viruses were incubated with MDCK cells in the presence of drugs, and CPE was determined by Neutral Red assay. Synergy volumes are plotted for each double combination, and for the triple combination as a function of increasing concentration of each drug as the third drug. Gray bars, A/California/04/09 (CA04); black bars, A/California/05/09 (CA05); hatched bars, A/California/10/09 (CA10). (A) Double combinations of amantadine/oseltamivir carboxylate (AMT/OSC), amantadine/ribavirin (AMT/RBV), and ribavirin/oseltamivir carboxylate (RBV/OSC). Triple combination of amantadine, ribavirin, and oseltamivir carboxylate as a function of (B) amantadine concentration, (C) ribavirin concentration, and (D) oseltamivir carboxylate concentration. Data are presented as the mean between 18 replicates from 6 experiments with 95% confidence intervals. The concentrations of each drug used in double and triple combinations are provided in Table S1. *P<0.05 versus double combination without drug.

We next assessed the synergistic activity of double and triple combinations of amantadine, ribavirin, and oseltamivir carboxylate over a range of concentrations of each drug against each 2009 H1N1 isolate. A quantitative measure of the total synergy (or antagonism) of a drug combination can be expressed in terms of synergy volumes, which represents the cumulative synergy and antagonism across all concentrations for all the drugs in a combination. Based on the empirically determined criterion of synergy volume >100 µg/mL^2^% using the lower confidence interval, all three double combinations were additive against the 2009 H1N1viruses (Table 1). By contrast, the TCAD regimen was synergistic against all three viruses over multiple concentrations of all three drugs, with the synergy occurring at 0.1 µg/mL and above for amantadine, 0.32 µg/mL and above for ribavirin, and 0.0032 µg/mL and above for oseltamivir carboxylate (Table 1). These data show that the synergy of the TCAD regimen occurred at clinically achievable concentrations for all three drugs, given that the expected average plasma concentrations based on the recommended doses are 0.43 µg/mL for amantadine, 1.3 µg/mL for ribavirin, and 0.3 µg/mL for oseltamivir carboxylate. Furthermore, the synergy of the TCAD regimen was greater than the synergy of any double combination tested for all three 2009 H1N1 strains. Figure 1B–1D shows the synergy of the TCAD regiment as a function of increasing concentrations of amantadine, ribavirin, or oseltamivir carboxylate as the third drug, representing the contribution of each drug to the synergy of the double combination without the third drug. These data reveal that the addition of each drug as the third drug to the double combinations resulted in a concentration-dependent increase in synergy, indicative that each drug contributed to the synergy of the TCAD regimen.

**Table 1:** Synergy volume of double and triple combinations of antivirals against 2009 H1N1 viruses A/California/04/09 (CA04), A/California/05/09 (CA05), and A/California/10/09 (CA10) as determined by Neutral Red assay.

Regimen	CA04	CA05	CA10
**Double combinations (µg/mL^2^%)**			
Zanamivir/Oseltamivir carboxylate	−24±116	−155±89	−197±98
Zanamivir/Peramivir	−35±112	−197±108	**−239±93**
Amantadine/Oseltamivir carboxylate	112±154	133±80	135±67
Amantadine/Ribavirin	12±82	22±36	69±59
Ribavirin/Oseltamivir carboxylate	178±188	222±143	166±73
**TCAD at fixed concentrations of amantadine (µg/mL^3^%)**			
0.1 µg/mL amantadine	106±208	**312±195**	**226±84**
0.32 µg/mL amantadine	292±212	**293±154**	**296±82**
1.0 µg/mL amantadine	**346±212**	**337±196**	**311±139**
3.2 µg/mL amantadine	**475±221**	**485±97**	**413±163**
**TCAD at fixed concentrations of ribavirin (µg/mL^3^%)**			
0.1 µg/mL ribavirin	92±119	146±82	171±137
0.32 µg/mL ribavirin	114±250	135±95	**271±105**
1.0 µg/mL ribavirin	179±292	**478±138**	**353±108**
3.2 µg/mL ribavirin	**691±144**	**763±256**	**632±216**
**TCAD at fixed concentrations of oseltamivir carboxylate (µg/mL^3^%)**			
0.001 µg/mL oseltamivir carboxylate	66±92	104±53	153±84
0.0032 µg/mL oseltamivir carboxylate	69±285	**248±93**	193±101
0.01 µg/mL oseltamivir carboxylate	**359±256**	**583±122**	**442±153**
0.032 µg/mL oseltamivir carboxylate	**465±291**	437±355	**317±164**

Synergy volumes represent the sum of the synergy or antagonism across all concentrations of two drugs for a double combination, or all concentrations of two drugs at a fixed concentration of the third drug for the TCAD regimen. The concentrations of each drug used are provided in Table S1. Combinations with synergy volumes >100 µg/mL^2^% for double combinations or >100 µg/mL^3^% for triple combinations are considered to be strongly synergistic (using the lower 95% confidence interval). Conversely, combinations with synergy volumes <−100 µg/mL^2^% or µg/mL^3^% are considered to be strongly antagonistic. Bold numbers denote volumes that are defined as strongly synergistic or antagonistic. Synergy volumes are presented as the mean between replicates with 95% confidence intervals. Typically, between 3 and 6 experiments were run for each combination, with each experiment having 3 replicate wells for each condition.

Importantly, despite the fact that amantadine had no significant antiviral activity as a single agent below 3.2 µg/mL (data not shown), we find that the amantadine contributed to the activity of the TCAD regimen at clinically achievable concentrations (0.43 µg/mL). Statistical analysis of the variability across all replicates from the six experiments for each virus revealed that amantadine made a significant contribution to the synergy of the TCAD regimen at concentrations 0.1 µg/mL and 0.32 µg/mL and above against CA05 and CA10, respectively, compared to the double combination of ribavirin/oseltamivir carboxylate without amantadine (Figure 1B). For CA04, while only the 3.2 µg/mL amantadine concentration had statistically significant greater synergy volume than the double combination without amantadine, there was a trend toward increasing synergy volume starting at 0.32 µg/mL. Thus, amantadine contributed to the activity of the TCAD regimen at concentrations where it was inactive as a single agent, and at concentrations that were clinically achievable.

Synergy plots, which reveal the extent of synergy at each concentration of each drug in the combinations, are shown in Figures S1 for CA05. The data are presented as contour plots, in which regions where inhibition is greater (synergy) or less (antagonism) than expected are identified by subtracting the theoretical additive inhibition from the observed inhibition. Synergy plots for TCAD regimen showed a concentration-dependent increase in synergy with respect to amantadine (Figure S1B, top plane). At the highest concentration tested (3.2 µg/mL), synergy was observed over a wide range of concentrations of ribavirin and oseltamivir carboxylate tested. At lower concentrations of amantadine, synergy occurred at 1 µg/mL ribavirin and higher, and at 0.0032 µg/mL oseltamivir carboxylate and higher. No significant antagonism was observed at any dose of any drug in the double combinations or the TCAD regimen. Similar patterns of synergy were observed with double and triple combinations of these antiviral drugs against CA04 and CA10 (data not shown).

### Enhancement of Antiviral Drug Activity in Triple Combination against 2009 H1N1

One notable consequence of synergy is that the antiviral activities of each drug in the combination is enhanced compared to the activities of the drugs as single agents. To demonstrate this, we compared the antiviral activity of each of the three drugs – amantadine, ribavirin, and oseltamivir carboxylate – as single agents and in the presence of fixed concentrations of the second and third drugs against CA10 replication. For each drug, the EC_50_ was reduced in triple combination compared to the EC_50_ as a single agent, indicative that each drug was active at lower concentrations (greater potency). For example, the EC_50_ for amantadine as a single agent was reduced by 3.2-fold in combination with 1 µg/mL ribavirin and 0.0032 µg/mL oseltamivir carboxylate; the EC_50_ for ribavirin as a single agent was reduced by 2.7-fold in combination with 0.0032 µg/mL oseltamivir carboxylate and 1 µg/mL amantadine; and the EC_50_ for oseltamivir carboxylate as a single agent was reduced 16.2-fold in combination with 1 µg/mL ribavirin and 1 µg/mL amantadine (Table 2). For all three drugs, the reduction in EC_50_ values observed in the triple combination compared to the single agent was statistically significant (*P*<0.05), indicative that each drug had greater antiviral potency and was effective at lower concentrations. In addition, the EC_50_ of all three drugs in triple combination were reduced 1.5- to 6.5-fold compared to the EC_50_ in double combinations, indicative of that the antiviral activity of the drugs in triple combination were enhanced compared to double combinations.

**Table 2:** The 50% effective concentration (EC_50_) of amantadine, ribavirin, and oseltamivir carboxylate as single agents and in double and triple combinations against 2009 H1N1 A/California/10/09 (CA10) as determined by Neutral Red assay.

Test Article	EC_50_ (µg/mL)	95% Confidence interval	Fold Reduction in EC_50_ Compared to (A)	Fold Reduction in EC_50_ Compared to (B)	Fold Reduction in EC_50_ Compared to (C)	P-values Compared to (A)	P-values Compared to (B)	P-values Compared to (C)
**Amantadine**								
(A) alone	24	23–25	–	–	–	–	–	–
(B) with 1 µg/mL ribavirin	13	12–14	1.8	–	–	<0.0001	–	–
(C) with 0.0032 µg/mL oseltamivir carboxylate	12	11–14	2.0	–	–	<0.0001	–	–
(D) with 1 µg/mL ribavirin and 0.0032 µg/mL oseltamivir carboxylate	7.6	6.0–9.6	3.2	1.7	1.6	<0.0001	<0.0001	<0.0001
**Ribavirin**								
(A) alone	6.5	6.2–6.9	–	–	–	–	–	–
(B) with 0.0032 µg/mL oseltamivir carboxylate	3.5	3.2–3.9	1.9	–	–	<0.0001	–	–
(C) with 1 µg/mL amantadine	5.2	4.6–5.9	1.2	–	–	0.007	–	–
(D) with 0.0032 µg/mL oseltamivir carboxylate and 1 µg/mL amantadine	2.4	1.7–3.4	2.7	1.5	2.2	<0.0001	0.0143	<0.0001
**Oseltamivir carboxylate**								
(A) alone	0.055	0.037–0.081	–	–	–	–	–	–
(B) with 1 µg/mL ribavirin	0.022	0.015–0.032	2.5	–	–	0.0009	–	–
(C) with 1 µg/mL amantadine	0.022	0.012–0.037	2.5	–	–	0.003	–	–
(D) with 1 µg/mL ribavirin and 1 µg/mL amantadine	0.0034	0.0009–0.012	16.2	6.5	6.5	<0.0001	0.0437	0.0015

EC_50_ values are the mean of five experiments (three replicates per experiment).

Importantly, the data presented here do not represent the maximum reductions in EC_50_ values for the three drugs. Due to the dynamic range of the assay, we were only able to obtain precise dose-response curves for each drug at fixed concentrations of the second and third drugs which were well below their EC_50_ values and well below concentrations where maximum synergy occurred. At higher concentrations, the antiviral activity of the second and third drug contributed significantly to the inhibition, which decreased the linear range of the assay and reduced the accuracy of the curve-fitting (data not shown). A comprehensive assessment of the interaction of two or three drugs in combination requires the evaluation of multiple concentrations of each drug in order to quantify synergy over the entire dosing range, as was done in the section above.

### Synergy of Double Combinations of Neuraminidase Inhibitors against 2009 H1N1

We also evaluated the interactions of double combinations of neuraminidase inhibitors (NAIs) against 2009 H1N1. As shown in Table 1, the synergy volumes for the zanamivir/oseltamivir carboxylate were −24±116 µg/mL^2^%, −155±89 µg/mL^2^%, and −197±98 µg/mL^2^% against CA04, CA05, and CA10, respectively. These values suggest that the zanamivir/oseltamivir carboxylate combination was additive against these viruses. For the zanamivir/peramivir combination, synergy volumes were −35±112 µg/mL^2^%, −197±108 µg/mL^2^% and −239±93 µg/mL^2^% against CA04, CA05, and CA10, respectively, indicative of additivity to moderate antagonism. Consistent with these values, the synergy plots for the NAI double combinations against CA05 revealed regions of antagonism (red areas), which occurred at higher concentrations of zanamivir (0.01–0.1 µg/mL) and at variable concentrations of the second NAI (oseltamivir or peramivir, Figure S2). Similar results were found with the other two 2009 H1N1 strains. Furthermore, evaluation of the EC_50_ of each NAI in combination with a fixed concentration of a second NAI revealed that the antiviral activity of each drug was not enhanced in combination with a second drug (data not shown). The observation that double combinations of neuraminidase inhibitors were not synergistic is consistent with the fact that all three drugs are known to target the same enzyme, and all bind in the same substrate binding pocket in a similar manner [19].

### Activity of Amantadine in the Context of the TCAD Regimen against Seasonal and Avian Amantadine-Resistant Viruses

**Figure 2:**
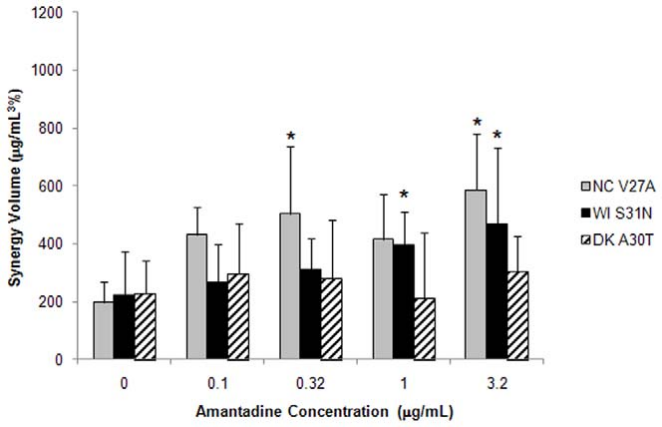
Synergy of the TCAD regimen against seasonal and avian amantadine-resistant viruses. Amantadine-resistant seasonal and avian viruses were incubated with MDCK cells in the presence of drugs, and CPE was determined by Neutral Red assay. Synergy volumes are plotted as a function of amantadine concentration, with 0 µg/mL amantadine being the ribavirin/oseltamivir carboxylate double combination. Gray bars, A/New Caledonia/20/99 (H1N1) V27A (NC V27A); black bars, A/Wisconsin/67/05 (H3N2) S31N (WI S31N); hatched bars, A/Duck/MN/1525/81 (H5N1) A30T (DK A30T). Data are presented as the mean between 10 replicates from 4 experiments with 95% confidence intervals for NC V27A, 27 replicates from 9 experiments with 95% confidence intervals for WI S31N, and 12 replicates from 4 experiments with 95% confidence intervals for DK A30T. The concentrations of each drug used in double and triple combinations are provided in Table S1. *P<0.05 versus double combination without amantadine.

As an extension of these studies we assessed the contribution of amantadine to the synergy of the TCAD regimen against other seasonal and avian amantadine-resistant viruses. The amantadine-resistant viruses tested included A/New Caledonia/20/99 (H1N1) bearing the V27A substitution in M2 (NC V27A), A/Wisconsin/67/05 (H3N2) bearing the S31N substitution in M2 (WI S31N), and A/Duck/MN/1525/81 (H5N1) bearing the A30T substitution in M2 (DK A30T). As a single agent, amantadine had no activity against these viruses at concentrations up to the highest concentration tested (32 µg/mL; data not shown). As shown in Figure 2, against NC V27A and WI S31N, amantadine contributed to the synergy of the TCAD regimen in a concentration-dependent manner. The synergy of the TCAD regimen in the presence of amantadine was greater than the synergy of the ribavirin/oseltamivir carboxylate double combination without amantadine, and the increase in the synergy of the TCAD regimen was observed at 0.32 µg/mL amantadine for NC V27A and 1 µg/mL amantadine for WI S31N, when significance was evaluated at the level of <0.05. Against DK A30T, however, amantadine did not make a contribution to the synergy of the TCAD regimen within the concentration range tested (0.1–3.2 µg/mL), and the synergy of the TCAD regimen was not greater than the synergy of the ribavirin/oseltamivir carboxylate double combination. Whether the lack of contribution from amantadine against the DK A30T virus was due to the specific subtype (H5N1), the M2 mutation (A30T), or the combination of both remain to be determined. However, it should be noted that while amantadine made no contribution to the activity of the TCAD regimen against DK A30T, both ribavirin and oseltamivir remain active and their interactions were additive, indicative that two out of three drugs contribute to the activity of the TCAD regimen against this virus (data not shown).

### Activity of Oseltamivir in the Context of the TCAD Regimen against Oseltamivir-Resistant Viruses

**Figure 3:**
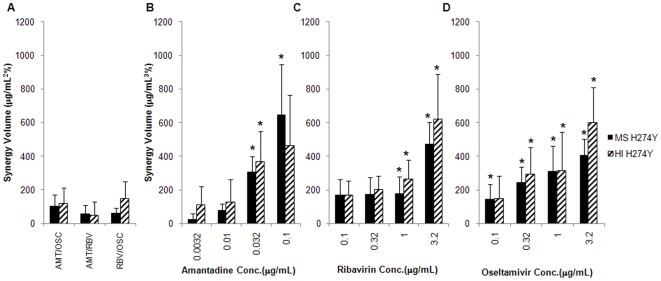
Synergy of double and triple combinations of amantadine, ribavirin, and oseltamivir carboxylate against oseltamivir-resistant H1N1. Oseltamivir-resistant H1N1 viruses were incubated with MDCK cells in the presence of drugs, and CPE was determined by Neutral Red assay. Synergy volumes are plotted for each double combination, and for the triple combination as a function of increasing concentration of each drug as the third drug. Black bars, A/Mississippi/3/01 H274Y (MS H274Y); hatched bars, A/Hawaii/21/07 H274Y (HI H274Y). (A) Double combinations of amantadine/oseltamivir carboxylate (AMT/OSC), amantadine/ribavirin (AMT/RBV), and ribavirin/oseltamivir carboxylate (RBV/OSC). Triple combination of amantadine, ribavirin, and oseltamivir carboxylate as a function of (B) amantadine concentration, (C) ribavirin concentration, and (D) oseltamivir carboxylate concentration. Data are presented as the mean between 18 replicates from 6 experiments with 95% confidence intervals. The concentrations of each drug used in double and triple combinations are provided in Table S1. *P<0.05 versus double combination without drug.

Given that a large percentage of seasonal influenza circulating viruses are resistant to oseltamivir, we next assessed the activity and synergy of the TCAD regimen against oseltamivir-resistant viruses to evaluate the spectrum of activity of the TCAD regimen, and to determine whether oseltamivir contributed to the activity of the TCAD regimen against oseltamivir-resistant viruses. Two H1N1 oseltamivir-resistant viruses were used, both of which bear the H274Y substitution in NA which has been demonstrated to confer resistance to oseltamivir [20]: A/Mississippi/3/01 (MS H274Y) and A/Hawaii/21/07 (HI H274Y). As a single agent, oseltamivir carboxylate had no antiviral activity against either virus below 1 µg/mL (data not shown). The synergy volumes of double and triple combinations of amantadine, ribavirin, and oseltamivir were determined against both viruses, and the data are presented in Figure 3. As double combinations, amantadine/oseltamivir carboxylate, amantadine/ribavirin, and ribavirin/oseltamivir carboxylate were all additive (Figure 3A). As was seen with the 2009 H1N1 viruses, the TCAD regimen was synergistic, and the synergy volume was greater than the synergy volume of any double combination, with each drug making a contribution to the synergy of the TCAD regimen against the oseltamivir-resistant viruses (Figure 3B–D). Importantly, oseltamivir carboxylate contributed to the synergy of the TCAD regimen starting at 0.1 µg/mL against MS H274Y (*P*<0.05) and at 0.32 µg/mL against HI H274Y (*P*<0.01). At these concentrations, which are achievable clinically, oseltamivir carboxylate is not active as a single agent against these resistant strains. Synergy plots for the TCAD regimen against MS H274Y and HI H274Y are provided in Figure S3, and reveal increasing synergy with increasing concentrations of oseltamivir carboxylate. At the highest concentration of oseltamivir carboxylate tested (3.2 µg/mL), synergy occurred over wide concentrations of ribavirin and amantadine. At lower concentrations of oseltamivir carboxylate, synergy occurred at higher concentrations of amantadine (0.1 µg/mL or higher), and at wide concentrations of ribavirin. No significant antagonism was detected at any concentrations of the three drugs.

We also determined the EC_50_ of oseltamivir carboxylate as a single agent and in double and triple combinations against both oseltamivir-resistant viruses. As summarized in Table 3, the EC_50_ of oseltamivir carboxylate as a single agent was 74 µg/mL against MS H274Y and 15 µg/mL against HI H274Y. These represent 1480- and 300-fold reductions in susceptibility compared to the published values for a wild-type virus [11]. The EC_50_ of oseltamivir carboxylate was not reduced in double combination with 1µg/mL ribavirin or 0.032 µg/mL amantadine against MS H274Y, and was reduced by only 1.7- and 1.4-fold, respectively, against HI H274Y. However, in triple combination with ribavirin and amantadine at the same concentrations used in double combination, the EC_50_ of oseltamivir carboxylate was reduced by 21-fold against MS H274Y and by 5.8-fold against HI H274Y.

**Table 3:** The 50% effective concentration (EC_50_) of oseltamivir carboxylate as single agents and in double and triple combinations against seasonal oseltamivir-resistant viruses as determined by Neutral Red assay.

Regimen	EC_50_ (µg/mL)	95% Confidence interval	Fold Reduction in EC_50_ Compared to (A)	Fold Reduction in EC_50_ Compared to (B)	Fold Reduction in EC_50_ Compared to (C)	P-values Compared to (A)	P-values Compared to (B)	P-values Compared to (C)
**Against A/Mississippi/3/01 (H1N1) H274Y**								
(A) oseltamivir carboxylate alone	73	46–114	–	–	–	–	–	–
(B) with 1.0 µg/mL ribavirin	>100	–	–	–	–	0.1127	–	–
(C) with 0.032 µg/mL amantadine	>100	–	–	–	–	0.0920	–	–
(D) with 1 µg/mL ribavirin and 0.032 µg/mL amantadine	3.4	1.2–9.6	21	>29	>29	<0.001	<0.0001	<0.0001
**Against A/Hawaii/21/07 (H1N1) H274Y**								
(A) oseltamivir carboxylate alone	15	9–23	–	–	–	–	–	–
(B) with 1 µg/mL ribavirin	8.7	5–14	1.7	–	–	0.1383	–	–
(C) with 0.032 µg/mL amantadine	11	5–25	1.4	–	–	0.5379	–	–
(D) with 1 µg/mL ribavirin and 0.032 µg/mL amantadine	2.6	1.1–6.3	5.8	3.3	4.2	0.0010	0.0189	0.0218

EC_50_ values are the mean of five experiments (three replicates per experiment).

### Cytotoxicity of Antiviral Drugs as Single Agents and in Double and Triple Combinations

The TC_50_ of amantadine as a single agent was 37–40 µg/mL, whereas the TC_50_ of ribavirin and oseltamivir carboxylate as single agents were >100 µg/mL (Table S2). These values are more than 10-fold higher than the highest concentration of each drug used in the combination experiments (Table S1). Furthermore, synergy analysis of the double and triple combinations revealed no synergistic cytotoxicity for any double combination or the TCAD regimen within the concentration ranges tested (data not shown). For example, cells treated with the TCAD regimen at the highest concentrations tested for all three drugs used in the combination experiments (3.2 µg/mL amantadine, 10 µg/mL ribavirin, and 3.2 µg/mL oseltamivir carboxylate) exhibited 97% viability, which was not statistically different than the cell control (P = 0.47 as determined by Student's t-test, Figure S4).

### Inhibitory Quotients of Antiviral Drugs against Susceptible and Resistant Viruses

One indicator of the expected clinical antiviral activity is the inhibitory quotient (IQ), defined herein as the ratio between the average total plasma concentration (C_ave_) and the 50% effective inhibitory concentration (EC_50_). In order to predict the effectiveness of the TCAD regimen against the circulating influenza strains in the clinical setting, we calculated and compared the IQs for amantadine, ribavirin, and oseltamivir carboxylate as single agents and the TCAD regimen against susceptible and resistant influenza viral strains (including 2009 H1N1). To determine the IQ of the TCAD regimen, amantadine, ribavirin, and oseltamivir carboxylate was tested as a fixed ratio combination, wherein the ratio of the three drugs was kept constant even as the total concentration of drugs varied. A dilution curve of the TCAD regimen was created by first preparing a solution of all three drugs at 100-fold the C_ave_ of each drug (43 µg/mL amantadine, 130 µg/mL ribavirin, 30 µg/mL oseltamivir carboxylate), and then serially diluting this solution in half-log_10_ increments. In this manner, the EC_50_ of the TCAD regimen was determined as a ratio of the C_ave_ and expressed in units of fold change from C_ave_.

As representatives of the current circulating strains, we used A/New Caledonia/20/99 (H1N1) (NC20) as the susceptible virus (susceptible to amantadine, ribavirin, and oseltamivir carboxylate), CA10 as the amantadine-resistant virus, and MS H274Y as the oseltamivir-resistant virus. Concentration-response curves for amantadine, ribavirin, oseltamivir carboxylate, and TCAD were generated against all three viruses, and the EC_50_s and IQs are summarized in Table 4. Amantadine was effective at inhibiting NC20 and MS H274Y *in vitro*, resulting in IQs of 1.95 and 7.17, respectively. However, the IQ for amantadine was reduced to 0.02 when tested against CA10, indicative that an in vitro concentration representing the achievable plasma concentration at the recommended dose was not adequate to inhibit the amantadine-resistant virus *in vitro*. An IQ of 0.02 represents a 100-fold reduction compared to NC20 and a 350-fold reduction compared to MS H274Y. Similarly, oseltamivir carboxylate was effective at inhibiting NC20 and CA10 *in vitro* resulting in IQs of 1.5 and 5.0, respectively, but not MS H274Y (IQ = 0.004). Similar to amantadine against the amantadine-resistant virus, the IQ for oseltamivir was reduced 350- to 2300-fold against the oseltamivir-resistant virus compared to susceptible viruses. The IQs for ribavirin were uniformly low and below 1 for all three viruses (0.23 to 0.42). On the other hand, the IQs for the TCAD regimen as a fixed dose combination were consistently high against all three viruses (8.33 to 17.24), varying by no more than 2-fold between susceptible and resistant viruses. These data suggest that the TCAD regimen may have broad utility against all circulating influenza strains, including strains that are resistant to either amantadine or oseltamivir.

**Table 4:** The 50% effective concentrations (EC_50_) and inhibitory quotients (IQ) for amantadine, ribavirin, oseltamivir carboxylate, and TCAD^a^ against representative susceptible and resistant viruses.

Strain	Regimen	EC_50_ (µg/mL)	95% Confidence Interval	EC_50_ (fold C_ave_)^b^	95% Confidence Interval	IQ (C_ave_/EC_50_)
**A/New Caledonia/20/99 (H1N1) susceptible**	Amantadine	0.22	0.13–0.39	–	–	1.95
	Ribavirin	3.1	2.1–4.5	–	–	0.42
	Oseltamivir carboxylate	0.20	0.14–0.28	–	–	1.50
	TCAD	–	–	0.058	0.045–0.074	17.24
**A/California/10/09 (H1N1) S31N amantadine-resistant**	Amantadine	20	16–24	–	–	0.02
	Ribavirin	3.2	01.6–4.8	–	–	0.41
	Oseltamivir carboxylate	0.032	0.30–0.34	–	–	9.38
	TCAD	–	–	0.065	0.044–0.095	15.38
**A/Mississippi/3/01 (H1N1) H274Y oseltamivir-resistant**	Amantadine	0.060	0.047–0.073	–	–	7.17
	Ribavirin	3.6	2.8–4.6	–	–	0.36
	Oseltamivir carboxylate	73	46–114	–	–	0.004
	TCAD	–	–	0.12	0.085–0.17	8.33

EC_50_ values are the mean of three to five experiments (three replicates per experiment).

a TCAD, triple combination antiviral drug (amantadine, ribavirin, and oseltamivir carboxylate) as a fixed-ratio combination.

b TCAD was formulated as a fixed-ratio combination based on the C_ave_ of each drug, wherein at 1-fold C_ave_ each drug was present at the following concentration: 0.43µg/mL amantadine, 0.3 µg/mL oseltamivir carboxylate, and 1.3 µg/mL ribavirin. TCAD was then titrated keeping the ratio of the drugs constant. The EC_50_ of TCAD is expressed as a ratio to the C_ave_.

## Discussion

Given that virtually all seasonal H3N2 and 2009 H1N1 strains are resistant to amantadine, and virtually all currently circulating seasonal H1N1 strains are resistant to oseltamivir, the pharmacologic rationale for the development of a triple combination antiviral drug (TCAD) composed of amantadine, ribavirin, oseltamivir is that at least two, and possibly three drugs, in the TCAD regimen will be active against all of these viruses. A number of studies have evaluated double combinations of antivirals [21]–[27] against influenza A infection *in vitro*, and Hayden et al. have tested a triple combination of two antivirals with human interferon α [28]. However, there have been few reports on the effects of drug combinations on resistant influenza viruses [26], [27], [29]. Recently, Smee et al. evaluated the effects of double combinations of amantadine, ribavirin, and oseltamivir against the same amantadine-resistant H5N1 virus used in this study (A/Duck/MN/1525/81) [26]. The authors found that the presence of amantadine in double combinations did not provide added benefit over the second drug alone, either in cell culture or in mouse models.

In the present study, we examined the efficacy and synergy of the TCAD regimen against viruses which were resistant to oseltamivir or amantadine, including 2009 H1N1. Consistent with the previous findings [9], we found that the 2009 H1N1 strains were susceptible to NAIs (oseltamivir carboxylate, zanamivir, and peramivir) and ribavirin, but were resistant to adamantanes (amantadine and rimantadine). Surprisingly, we found that amantadine as a single agent retained partial activity against these viruses (Table S2), albeit the activity was reduced by 100-fold compared to a susceptible virus, whereas rimantadine had no activity below the 50% cytotoxic concentration. This observation suggests that phenotypic testing, in addition to determination of the genotype, may be necessary in order to fully understand the susceptibility profile of a novel virus and may have important implications in guiding the choice of antivirals for use in combinations.

Against the 2009 H1N1 strains, the interactions of oseltamivir carboxylate, peramivir, and zanamivir in double combinations ranged from additive to moderately antagonistic, indicative that the activity of these drugs was not enhanced in combination compared to their activity as single agents. These results suggest that double combinations of NAIs may not provide any added benefit over the drugs as single agents. Given that all NAIs bind in the same substrate binding pocket in NA, the use of these drugs in combination in the absence of enhanced activity raises the risk of selecting for a single mutation that could confer resistance to both neuraminidase inhibitors simultaneously. Indeed, cross-resistance to oseltamivir and zanamivir resulting from a single amino acid change has been documented for seasonal influenza A and B viruses [30]. If this were to occur in the 2009 H1N1 background, the resulting virus would be resistant to all approved anti-influenza drugs.

In total, we tested the activity and synergy of the TCAD regimen against six amantadine-resistant viruses, including three strains of 2009 H1N1, and two oseltamivir-resistant viruses. The viruses tested in this study come from the three subtypes that cause significant morbidity and mortality in humans (H1N1, H3N2, and H5N1), and include seasonal, avian, and pandemic strains. The double combinations of amantadine/oseltamivir carboxylate, amantadine/ribavirin, and ribavirin/oseltamivir carboxylate were additive against 2009 H1N1, and ranged from additive to moderately synergistic against the other viruses (data not shown). In contrast, with the exception of the duck H5N1 virus, we found that the TCAD regimen was synergistic at clinically achievable concentrations of all three drugs, and that the synergy of the TCAD regimen was greater than that of any double antiviral drug combination. These data suggest that the TCAD regimen may have broad-spectrum antiviral activity against circulating influenza A viruses, including strains that are resistant to either classes of antivirals. To date, most influenza A strains in circulation (∼99%) are resistant to either the adamantanes or oseltamivir, and not to both [31], and thus are expected to be susceptible to the TCAD regimen. Currently, rapid diagnostic tests are not available to determine the susceptibility profile of influenza viruses in real time, and thus clinicians do not often have the necessary information with which to guide appropriate antiviral use. The availability of a broad-spectrum antiviral therapy that would be effective against the majority of circulating strains regardless of the susceptibility would be of high clinical utility.

Importantly, we found that amantadine and oseltamivir contributed to the synergy of the TCAD regimen against amantadine-resistant and oseltamivir-resistant viruses. The contributions from both drugs to the synergy of the TCAD regimen were significant at clinically achievable concentrations where they had little or no antiviral activity as a single agent. For instance, a comparison of the synergy volume of the TCAD regimen at 0.32 µg/mL amantadine to the synergy volume of the ribavirin/oseltamivir carboxylate double combination (no amantadine) revealed that amantadine contributed 39%, 24%, and 44% to the total synergy of the TCAD regimen against CA04, CA05, and CA10, respectively. Similarly, against the oseltamivir-resistant viruses, oseltamivir carboxylate at 0.32 µg/mL contributed 76% and 83% to the total synergy of the TCAD regimen against MS H274Y and HI H274Y, respectively. Thus, all three drugs contributed to the synergy and activity of the TCAD regimen against amantadine- and oseltamivir-resistant viruses, and the activities of amantadine and oseltamivir were restored in the context of the TCAD regimen against influenza strains that were resistant to these drugs, thereby maximizing the clinical utility of these drugs.

The mechanism(s) by which amantadine and oseltamivir carboxylate contribute to the synergy of the TCAD regimen against resistant strains is unclear. The interactions between M2, HA, and NA on the surface of the influenza particle are complex and not well understood, and a number of studies have demonstrated that HA-M2 and HA-NA interactions can affect the susceptibility to amantadine and oseltamivir, respectively [32], [33]. Furthermore, amantadine has been demonstrated to exert antiviral activity via interactions with HA at higher concentrations [34], [35]. It is conceivable that, as the result of protein-protein interactions between M2, HA, and NA, the binding of a drug at one site may affect the conformation and therefore affinity for another drug at another site. The mechanism by which ribavirin contributes to the synergy of the TCAD regimen is also unclear. Ribavirin has been documented to act through multiple mechanisms affecting both virus replication and host immune response [36]–[38], and it remains to be elucidated which of these mechanisms are responsible for the synergy with amantadine and oseltamivir.

Finally, we evaluated the activity and inhibitory quotient (IQ) of TCAD against susceptible and resistant viruses representing the currently circulating strains. While the correlation between IQ and clinical efficacy has not been demonstrated for influenza, it is valuable to construct a relative ranking of the IQ of different antiviral regimens against susceptible and resistant viruses in order to assess the spectrum of their activity. When tested against a seasonal susceptible H1N1 virus, an amantadine-resistant 2009 H1N1, and a seasonal oseltamivir-resistant H1N1 virus, TCAD was uniformly active against all three viruses with significantly high IQs (8.33 to 17.24; Table 4). This suggests that TCAD may have broad antiviral activity against all currently circulating influenza strains and may have good efficacy in the clinical setting against these strains.

Our data suggests that a triple combination antiviral drug (TCAD) composed of amantadine, ribavirin, and oseltamivir may be an effective and viable therapeutic option for the treatment of pandemic and seasonal influenza infection. The body of data presented in this report validates the TCAD hypothesis, which states that for any given susceptible or resistant circulating influenza virus, at least two, and in some cases all three, drugs in TCAD will be active. Furthermore, the TCAD regimen appears to overcome baseline drug resistance and thus may represent a highly active antiviral therapy for seasonal and pandemic influenza. The safety, pharmacokinetics, distribution, and metabolism of amantadine, ribavirin, and oseltamivir as single agents are well understood, and it is not expected that co-administration of the three drugs will result in substantially increased risk to patients compared to the administration of the individual drugs. In addition, all three double combinations have been tested in humans without adverse effects, including amantadine plus oseltamivir [39], amantadine plus ribavirin [40], and ribavirin plus oseltamivir [41]. Clinical trials to assess the efficacy and safety of TCAD for the treatment of influenza have been initiated, and will provide important data on the use of TCAD against both pandemic and seasonal influenza.

## Supporting Information

Table S1 Concentration ranges (µg/mL) of each drug tested in double and triple combinations against different influenza A viruses. Drugs were titrated at half log_10_ increments. NT, not tested. CA04, A/California/04/09 (H1N1); CA05, A/California/05/09 (H1N1); CA10, A/California/10/09 (H1N1); NC V27A, A/New Caledonia/20/99 (H1N1); WI S31N, A/Wisconsin/67/05 (H3N2); DK A30T, A/Duck/MN/1525/81 (H5N1); MS H274Y, A/Mississippi/3/01 (H1N1); HI H274Y, A/Hawaii/21/07 (H1N1).
(0.05 MB DOC)

Table S2 The 50% effective concentrations (EC_50_) with 95% confidence intervals (95% CI), and 50% cytotoxic concentrations (TC_50_) of different antiviral agents against 2009 H1N1 viruses. EC_50_ and TC_50_ values are the mean of at least 5 experiments (three replicates per experiment) as determined by Neutral Red assay. CA04, A/California/04/09; CA05, A/California/05/09; CA10, A/California/10/09. ^a^Rimantadine was not active up to the 50% cytotoxic concentration.
(0.06 MB DOC)

Figure S1 Synergy plot of double and triple combinations of amantadine, ribavirin, and oseltamivir carboxylate against 2009 H1N1 A/California/05/09 (CA05) replication as determined by Neutral Red assay in MDCK cells. Calculated additive interactions were subtracted from the experimentally determined inhibition to reveal regions of synergy (inhibition above expected) or antagonism (inhibition below expected). Values were derived from mean triplicate data and presented at 95% confidence. This experiment was repeated a total of six times with similar results. Blue areas indicate concentrations of each drug that are synergistic, gray areas indicate concentrations that are additive, and red areas indicate concentrations that are antagonistic. The intensity of the color (blue or red) corresponds to percent inhibition above or below expected. (A) Double combinations of amantadine/oseltamivir carboxylate (top); amantadine/ribavirin (middle); and ribavirin/oseltamivir carboxylate (bottom). Concentrations of each drug are indicated on the axes. (B) Triple combinations of amantadine, ribavirin, and oseltamivir carboxylate. Concentrations of each drug are indicated on the axes, with each plane representing a different concentration of amantadine.
(5.74 MB TIF)

Figure S2 Synergy plot of double combinations of zanamivir, oseltamivir carboxylate, and peramivir against 2009 H1N1 A/California/05/09 (CA05) replication as determined by Neutral Red assay in MDCK cells. Values were derived from mean triplicate data and presented at 95% confidence. This experiment was repeated a total of three times for the zanamivir/oseltamivir carboxylate combination and four times for the zanamivir/peramivir combination with similar results. Blue areas indicate concentrations of each drug that are synergistic, gray areas indicate concentrations that are additive, and red areas indicate concentrations that are antagonistic. Double combination of (A) zanamivir and oseltamivir carboxylate; and (B) zanamivir and peramivir. Concentrations of each drug are indicated on the axes.
(2.20 MB TIF)

Figure S3 Synergy of triple combinations of amantadine, ribavirin, and oseltamivir carboxylate against A/Mississippi/3/01(H1N1) H274Y (MS H274Y) and A/Hawaii/21/07 (H1N1) H274Y (HI H274Y) replication as determined by Neutral Red assay in MDCK cells. Values were derived from mean triplicate data and presented at 95% confidence. This experiment was repeated a total of six times with similar results. Blue areas indicate concentrations of each drug that are synergistic, gray areas indicate concentrations that are additive, and red areas indicate concentrations that are antagonistic. (A) MS H274Y; (B) HI H274Y. Concentrations of each drug are indicated on the axes, with each plane representing a different concentration of oseltamivir carboxylate.
(4.13 MB TIF)

Figure S4 Viability of MDCK cells treated with the TCAD regimen. MDCK cells were incubated with the TCAD regimen at the highest concentrations of all three drugs used in the synergy experiments (3.2 µg/mL amantadine, 10 µg/mL ribavirin, and 3.2 µg/mL oseltamivir carboxylate), and cell viability was determined by Neutral Red assay after 72 hours. Values are the mean of nine replicates from three experiments, with standard deviations. The difference in viability between the TCAD treated cells and the cell controls was not statistically significant (P = 0.47, Student's t-test).
(1.00 MB TIF)
