# Supplementary material for: Triple Combination of Amantadine, Ribavirin, and Oseltamivir Is Highly Active and Synergistic against Drug Resistant Influenza Virus Strains In Vitro
Source: PLoS One. 2010 Feb 22;5(2):e9332. doi: 10.1371/journal.pone.0009332 (PMC2825274; doi:10.1371/journal.pone.0009332)
Supplement: Table S1 — Concentration ranges (µg/mL) of each drug tested in double and triple combinations against different influenza A viruses. Drugs were titrated at half log10 increments. NT, not tested. CA04, A/California/04/09 (H1N1); CA05, A/California/05/09 (H1N1); CA10, A/California/10/09 (H1N1); NC V27A, A/New Caledonia/20/99 (H1N1); WI S31N, A/Wisconsin/67/05 (H3N2); DK A30T, A/Duck/MN/1525/81 (H5N1); MS H274Y, A/Mississippi/3/01 (H1N1); HI H274Y, A/Hawaii/21/07 (H1N1). (0.05 MB DOC) [file pone.0009332.s001.doc]

| Drug | CA04 | CA05 | CA10 | NC V27A | WI S31N | DK A30T | MS H274Y | HI H274Y |
| --- | --- | --- | --- | --- | --- | --- | --- | --- |
| **Amantadine** | 0, 0.1-3.2 | 0, 0.1-3.2 | 0, 0.1- 3.2 | 0, 0.1-3.2 | 0, 0.1- 3.2 | 0, 0.1-3.2 | 0, 0.001-0.032 | 0, 0.0032- 0.1 |
| **Ribavirin** | 0, 0.1- 10 | 0, 0.1- 10 | 0, 0.1-10 | 0, 0.032-3.2 | 0, 0.1-10 | 0, 0.1- 10 | 0, 0.1- 10 | 0, 0.1-10 |
| **Oseltamivir carboxylate** | 0, 0.001- 0.1 | 0, 0.001-0.1 | 0, 0.001- 0.1 | 0, 0.0032- 0.32 | 0, 0.00032- 0.032 | 0, 0.01- 1.0 | 0, 0.1-3.2 | 0, 0.1-3.2 |
| **Peramivir** | 0, 0.001- 0.1 | 0, 0.001- 0.1 | 0, 0.001- 0.1 | NT | NT | NT | NT | NT |
| **Zanamivir** | 0, 0.001- 0.1 | 0, 0.001- 0.1 | 0, 0.001- 0.1 | NT | NT | NT | NT | NT |
