# Supplementary material for: Triple Combination of Amantadine, Ribavirin, and Oseltamivir Is Highly Active and Synergistic against Drug Resistant Influenza Virus Strains In Vitro
Source: PLoS One. 2010 Feb 22;5(2):e9332. doi: 10.1371/journal.pone.0009332 (PMC2825274; doi:10.1371/journal.pone.0009332)
Supplement: Table S2 — The 50% effective concentrations (EC50) with 95% confidence intervals (95% CI), and 50% cytotoxic concentrations (TC50) of different antiviral agents against 2009 H1N1 viruses. EC50 and TC50 values are the mean of at least 5 experiments (three replicates per experiment) as determined by Neutral Red assay. CA04, A/California/04/09; CA05, A/California/05/09; CA10, A/California/10/09. aRimantadine was not active up to the 50% cytotoxic concentration. (0.06 MB DOC) [file pone.0009332.s002.doc]

|  | EC_50_ (µM) | EC_50_ (µg/mL) | 95% CI (µg/mL) | TC_50_ (µg/mL) |
| --- | --- | --- | --- | --- |
| Amantadine |  |  |  |  |
| CA04 | 85 | 16 | 12-20 | 39 |
| CA05 | 106 | 20 | 17-24 | 37 |
| CA10 | 106 | 20 | 19-22 | 40 |
| Rimantadine |  |  |  |  |
| CA04 | >51^a^ | >11^a^ | ND | 11 |
| CA05 | >55^a^ | >12^a^ | ND | 12 |
| CA10 | >51^a^ | >11^a^ | ND | 11 |
| Oseltamivir |  |  |  |  |
| CA04 | 0.18 | 0.058 | 0.03-0.084 | >100 |
| CA05 | 0.19 | 0.061 | 0.05-0.076 | >100 |
| CA10 | 0.09 | 0.030 | 0.026-0.035 | >100 |
| Zanamivir |  |  |  |  |
| CA04 | 0.35 | 0.12 | 0.025-0.21 | >100 |
| CA05 | 0.23 | 0.078 | 0.061-0.099 | >100 |
| CA10 | 0.13 | 0.043 | 0.037-0.049 | >100 |
| Peramivir |  |  |  |  |
| CA04 | 0.016 | 0.0053 | 0.0013-0.0094 | >100 |
| CA05 | 0.21 | 0.069 | 0.051-0.093 | >100 |
| CA10 | 0.13 | 0.043 | 0.032-0.058 | >100 |
| Ribavirin |  |  |  |  |
| CA04 | 28 | 7.0 | 3.3-10 | >100 |
| CA05 | 23 | 5.6 | 5.1-6.3 | >100 |
| CA10 | 13 | 3.2 | 2.8-3.6 | >100 |
