# Supplementary figures and images for: Triple Combination of Amantadine, Ribavirin, and Oseltamivir Is Highly Active and Synergistic against Drug Resistant Influenza Virus Strains In Vitro
Source: PLoS One. 2010 Feb 22;5(2):e9332. doi: 10.1371/journal.pone.0009332 (PMC2825274; doi:10.1371/journal.pone.0009332)

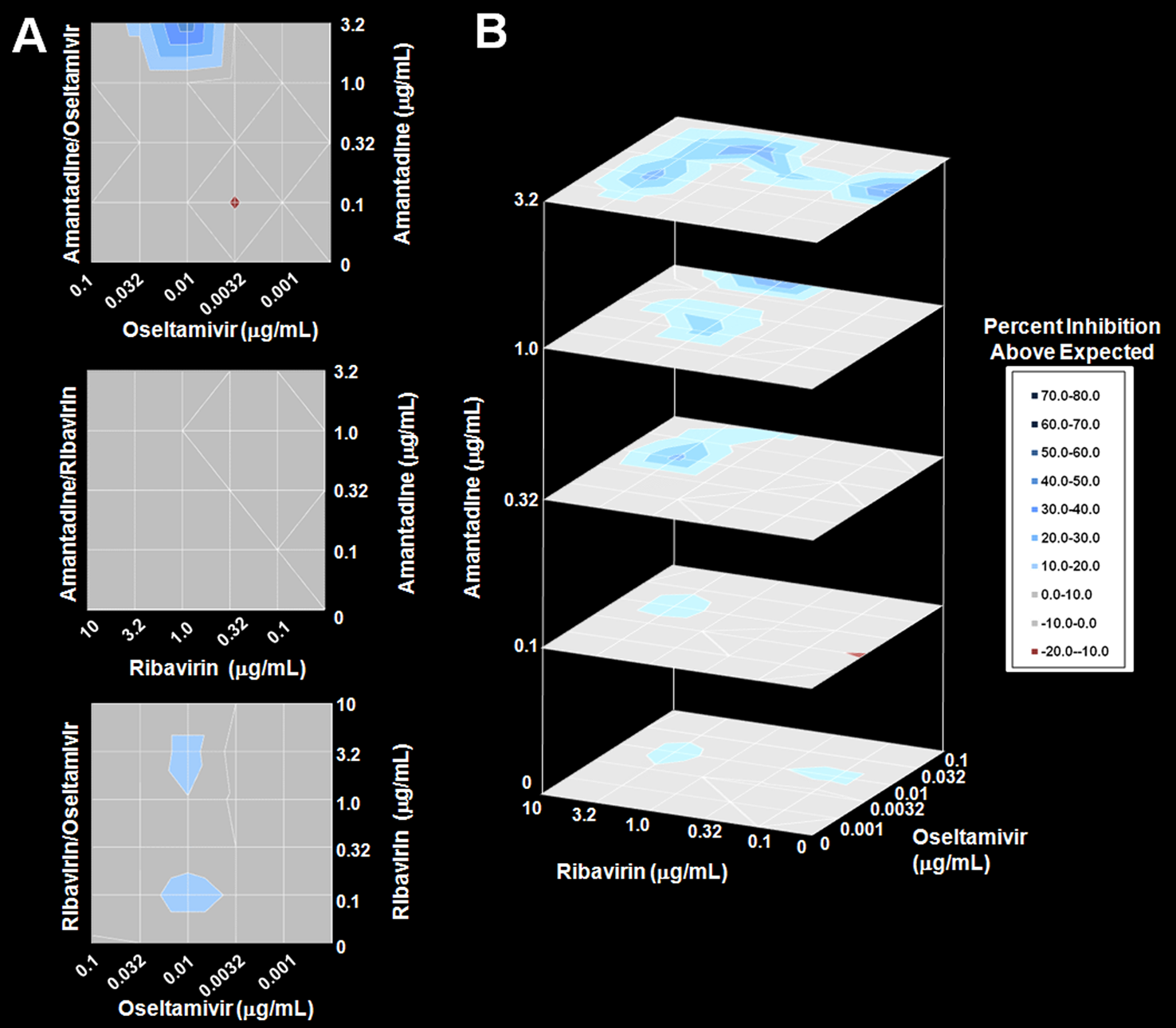

Supplement: Figure S1 — Synergy plot of double and triple combinations of amantadine, ribavirin, and oseltamivir carboxylate against 2009 H1N1 A/California/05/09 (CA05) replication as determined by Neutral Red assay in MDCK cells. Calculated additive interactions were subtracted from the experimentally determined inhibition to reveal regions of synergy (inhibition above expected) or antagonism (inhibition below expected). Values were derived from mean triplicate data and presented at 95% confidence. This experiment was repeated a total of six times with similar results. Blue areas indicate concentrations of each drug that are synergistic, gray areas indicate concentrations that are additive, and red areas indicate concentrations that are antagonistic. The intensity of the color (blue or red) corresponds to percent inhibition above or below expected. (A) Double combinations of amantadine/oseltamivir carboxylate (top); amantadine/ribavirin (middle); and ribavirin/oseltamivir carboxylate (bottom). Concentrations of each drug are indicated on the axes. (B) Triple combinations of amantadine, ribavirin, and oseltamivir carboxylate. Concentrations of each drug are indicated on the axes, with each plane representing a different concentration of amantadine. (5.74 MB TIF) [file pone.0009332.s003.tif]

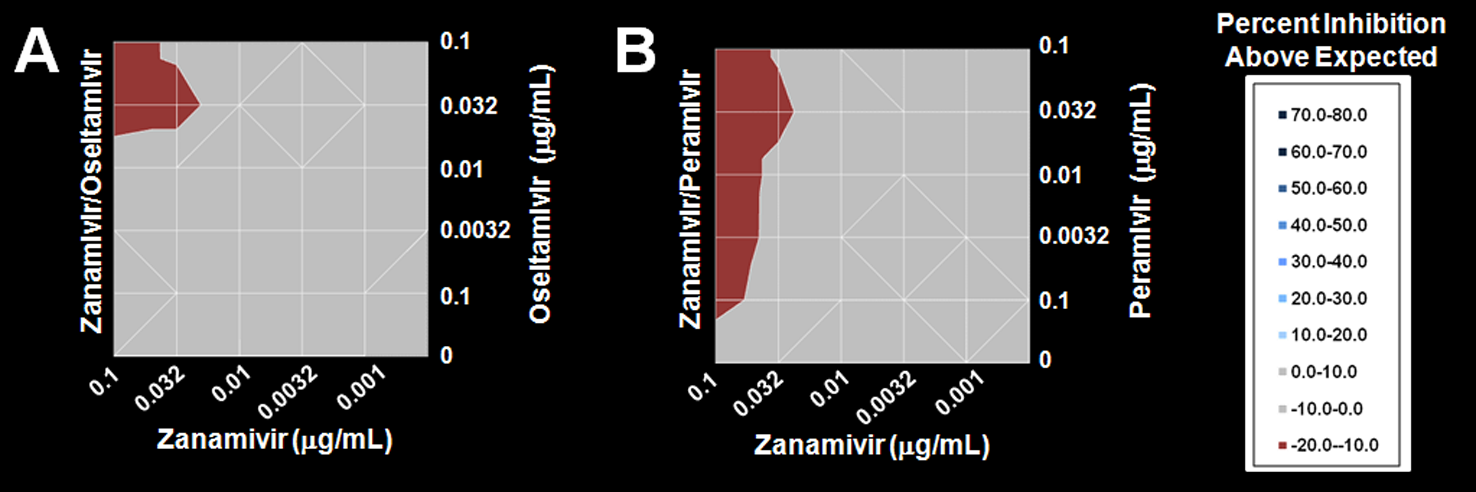

Supplement: Figure S2 — Synergy plot of double combinations of zanamivir, oseltamivir carboxylate, and peramivir against 2009 H1N1 A/California/05/09 (CA05) replication as determined by Neutral Red assay in MDCK cells. Values were derived from mean triplicate data and presented at 95% confidence. This experiment was repeated a total of three times for the zanamivir/oseltamivir carboxylate combination and four times for the zanamivir/peramivir combination with similar results. Blue areas indicate concentrations of each drug that are synergistic, gray areas indicate concentrations that are additive, and red areas indicate concentrations that are antagonistic. Double combination of (A) zanamivir and oseltamivir carboxylate; and (B) zanamivir and peramivir. Concentrations of each drug are indicated on the axes. (2.20 MB TIF) [file pone.0009332.s004.tif]

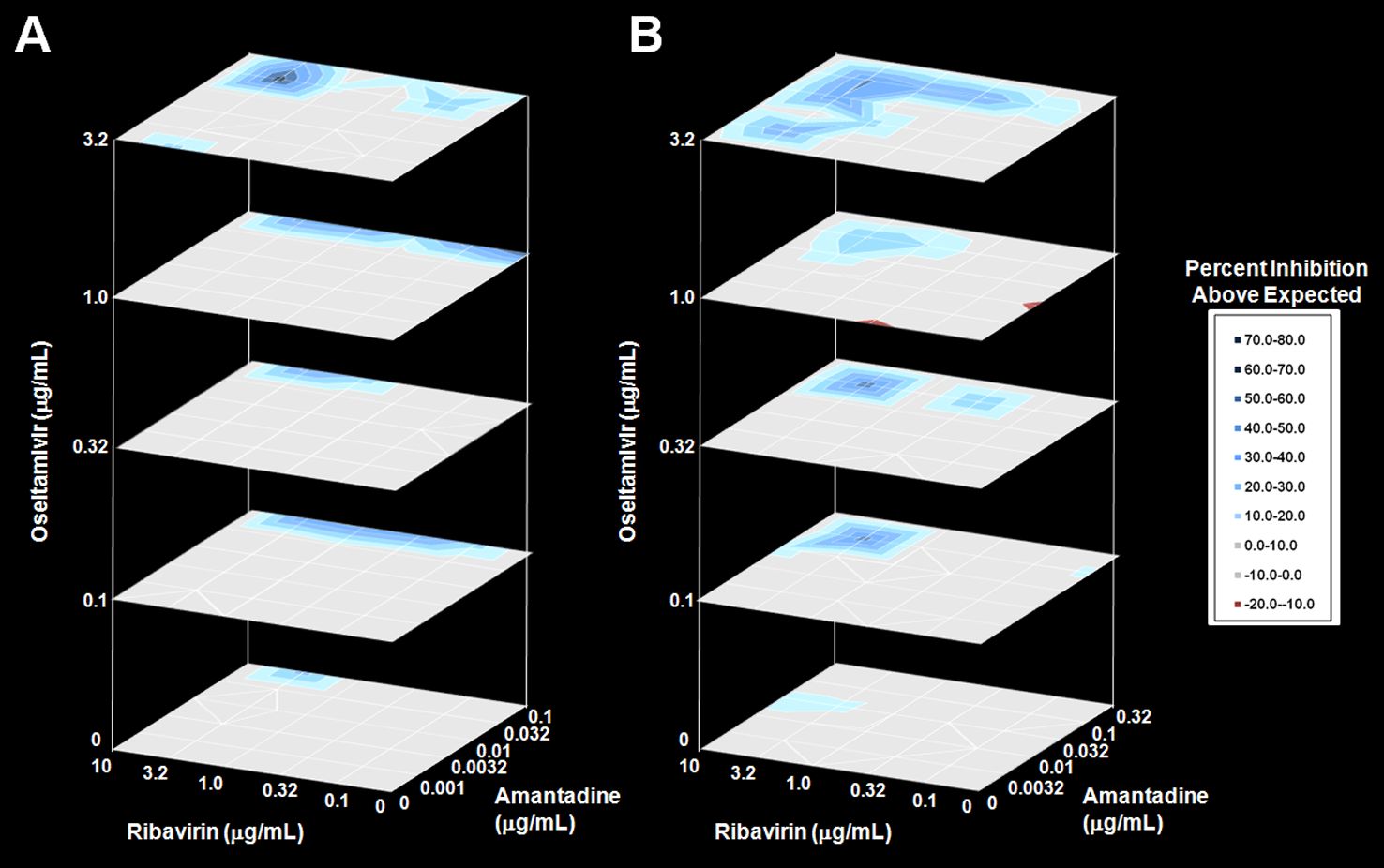

Supplement: Figure S3 — Synergy of triple combinations of amantadine, ribavirin, and oseltamivir carboxylate against A/Mississippi/3/01(H1N1) H274Y (MS H274Y) and A/Hawaii/21/07 (H1N1) H274Y (HI H274Y) replication as determined by Neutral Red assay in MDCK cells. Values were derived from mean triplicate data and presented at 95% confidence. This experiment was repeated a total of six times with similar results. Blue areas indicate concentrations of each drug that are synergistic, gray areas indicate concentrations that are additive, and red areas indicate concentrations that are antagonistic. (A) MS H274Y; (B) HI H274Y. Concentrations of each drug are indicated on the axes, with each plane representing a different concentration of oseltamivir carboxylate. (4.13 MB TIF) [file pone.0009332.s005.tif]

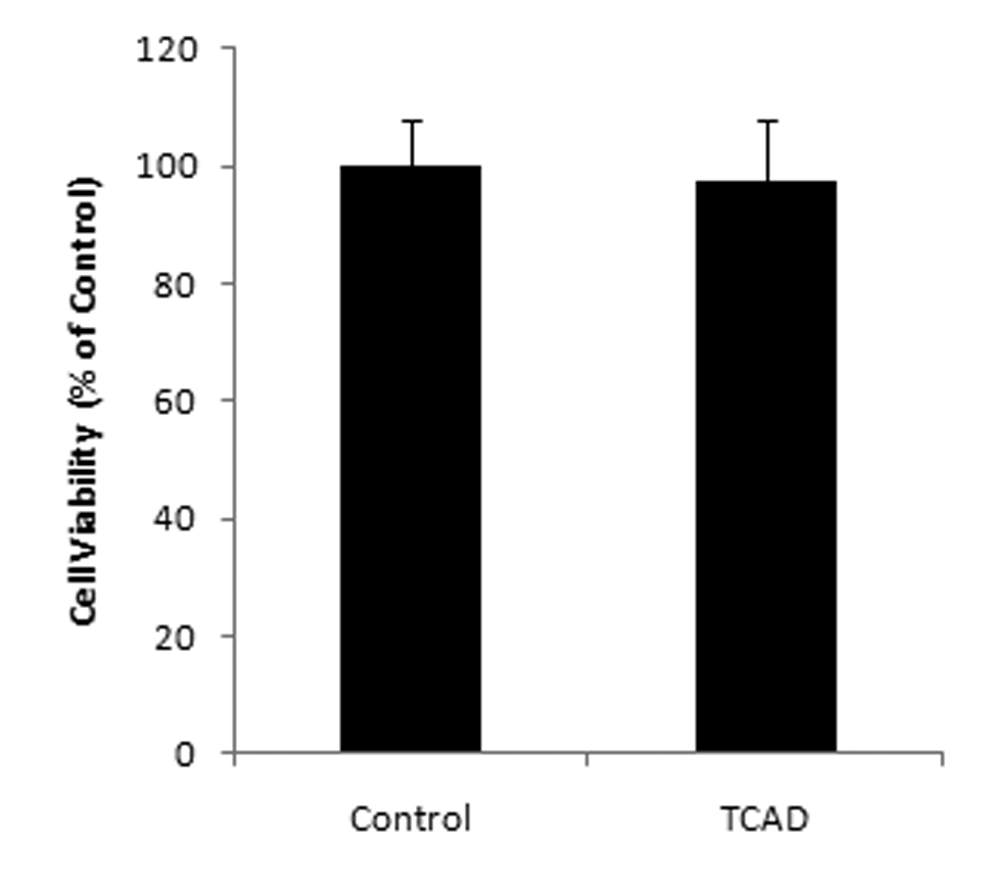

Supplement: Figure S4 — Viability of MDCK cells treated with the TCAD regimen. MDCK cells were incubated with the TCAD regimen at the highest concentrations of all three drugs used in the synergy experiments (3.2 µg/mL amantadine, 10 µg/mL ribavirin, and 3.2 µg/mL oseltamivir carboxylate), and cell viability was determined by Neutral Red assay after 72 hours. Values are the mean of nine replicates from three experiments, with standard deviations. The difference in viability between the TCAD treated cells and the cell controls was not statistically significant (P = 0.47, Student's t-test). (1.00 MB TIF) [file pone.0009332.s006.tif]
